# Prevalence of antenatal depression among women receiving antenatal care during last trimester of pregnancy in a tertiary care private institute of Lahore

**DOI:** 10.12669/pjms.35.2.649

**Published:** 2019

**Authors:** Mahrukh Sabir, Muhammad Luqman Farrukh Nagi, Tahseen Haider Kazmi

**Affiliations:** 1 *Dr. Mahrukh Sabir, MBBS, Department of Community Medicine, Shalamar Institute of Health Sciences, Lahore, Pakistan*; 2 *Dr. Muhammad Luqman Farrukh Nagi, MBBS, MPH(Australia), Department of Community Medicine, Shalamar Institute of Health Sciences, Lahore, Pakistan*; 3 *Dr. Tahseen Haider Kazmi, MBBS, MPH(Australia), FCPS, Department of Community Medicine, Shalamar Institute of Health Sciences, Lahore, Pakistan*

**Keywords:** Antenatal depression, Passive smoking, Goldberg Depression Scale, Third trimester

## Abstract

**Objective:**

Pregnancy and depression affect each other. The main objective of our study was to find out frequency of antenatal depression during last trimester and associated risk factors among pregnant female visiting a tertiary care private institution of Lahore, Pakistan.

**Methods:**

This study was conducted at Shalamar Hospital a private tertiary care institution of Lahore during May 2017 to June 2018. Assuming 18% prevalence with design effect 1.5 and 97% confidence interval the calculated sample size was 417. To avoid dropouts the questionnaire was administered to 450 pregnant females in their last trimester that is with gestational age above 28 weeks. In this study we used a modified version of Goldberg’s depression scale in Urdu language for data collection. Wherever needed the data collection team read out aloud the questionnaire to the participants who did not know how to read and write. Informed consent in a written form (in Urdu language) was taken from every study participant after explaining the participants about the research. If the participant did not know how to read and write, the interviewers narrated out aloud the script of informed consent for them and got it signed later by the participants. The confidentiality, anonymity and privacy of the study participants were preserved. Ethical approval of this study was obtained from Institutional Review Board of Shalamar Institute of Health Sciences. Data entry and analysis was finalized by using SPSS version 20.

**Results:**

The prevalence of depression among women seeking antenatal care during their last trimester according to Goldberg Depression Scale was 40.89% (n=184).Whereas, 30.4% (n=137), 8.67% (n=39) and 1.78% (n=8) suffered from mild, moderate and severe depression respectively. The associations between passive smoking (p < 0.01), family history of preeclampsia (p < 0.05) and depression among family members (p < 0.05) with antenatal depression during last trimester were significant.

**Conclusion:**

According to Goldberg Depression Scale, 40.89% (n=184) of pregnant women seeking antenatal care in private tertiary care institution of Lahore suffered from depression.

## INTRODUCTION

In pregnancy there are chief physiological as well as psychological episode.^1^ Pregnancy and depression influence each other.^2^ Depression is an affective disorder, it is also characterized by sustained sorrow and noticeable anhedonia in daily routine as chief symptoms prevailing for one or more than one week. Additional alarming signs are sense of worthlessness, feeling offended and irritable, sleeplessness, appetite variations, diminished energy, poor concentration, decreased memory, and feelings of suicidal attempt or abortion.^3^ Depression is a frequent psychological infirmity ranking the third most common disabling disorder universally by the World Health Organization.^4^ Antenatal depression can be a precursor of postpartum depression.^5^ Pregnant mothers with prenatal depression are less probable to attend antenatal assessment, which contributes to unfavorable pregnancy outcomes for example preterm birth, low birth weight and intrauterine growth retardation.^6^-^8^ Depressed pregnant women are more likely to suffer from obstetrical impediments like pre-eclampsia.^9^,^10^

The children of mother with depression may experience impaired neurological, cognitive, emotional, and behavioral growth.^11^ A large number of epidemiological studies have revealed that 10-15% of women of childbearing age show depressive symptoms.^11^,^12^ The highest prevalence estimates of antepartum suicidal ideation (23–33%) have been accounted in studies conducted in the United States.^13^ The prevalence of antenatal depression was 14.2% in Brazil,^14^ 19% in Jordan,15.5% in Malta^15^, 25% in Jamaica, more than 48.4% in Pakistan.^16^-^17^

There is scarcity of research to assess the antenatal depression among women seeking antenatal care in tertiary care private institutions of Lahore. The purpose of this study was to find out frequency of perinatal depression and its associated risk factors among women during pregnancy in a tertiary care private institution of Lahore, Pakistan.

## METHODS

Convenience sampling technique was used in this cross sectional study, conducted at Shalamar Hospital a private tertiary care institution of Lahore during May 2017 to June 2018 after Ethical Approval by Institutional Review Board of Shalamar Institute of Health Sciences. Karmaliani R et al. in 2009 screened all pregnant women living in identified areas of Hyderabad, Pakistan using a validated Aga Khan University Anxiety Depression Scale found out that 18% of the women were anxious and/or depressed.^18^ Assuming 18% prevalence of depression, with design effect 1.5 and at 97% confidence interval the calculated sample size was 417. To avoid dropouts the questionnaire was administered to 450 pregnant females in their last trimester that is with gestational age above 28 weeks. Data was collected through a close ended questionnaire based on Goldberg’s depression scale from expectant females after getting informed consent. Both informed consent and Goldberg’s depression scale were taken on the forms translated in Urdu language. Informed consent in a written form (in Urdu language) was taken from every study participant after explaining the participants about the research. If the participants did not know how to read and write, the interviewers narrated out aloud the script of informed consent for them and got it signed later by the participants. In this study we used a modified version of Goldberg’s depression scale translated in Urdu language for data collection. Wherever needed the data collection team read out aloud the questionnaire to the participants who did not know how to read and write. The confidentiality and privacy of the participants was preserved by coding of data. Goldberg’s depression scale is a validated tool used for screening of depression. Based on the scores, the participants who scored above 21 were counseled and referred to the psychiatry department for management. Data entry and analyses were done in SPSS for Windows version 20. All results were regarded statistically significant having p value less than 0.05.

## RESULTS

**Fig.1:**
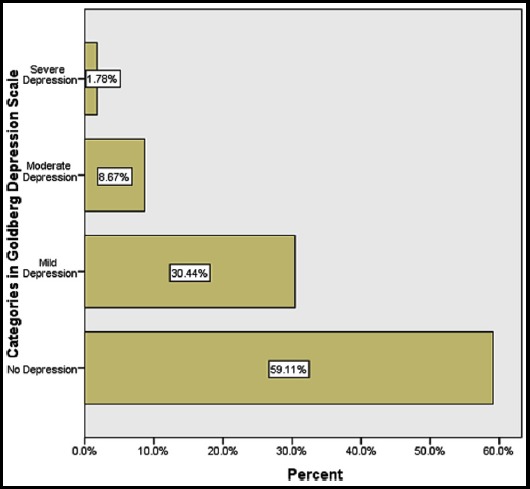
Frequency of depression among pregnant females in last trimester of pregnancy according to Goldberg Depression Scale (n=450).

The response rate was 100% (n= 450). The mean age of study participants was 27.5 ±4.3. The average height in inches was 61.8±2.4. Pre-pregnancy weight in kilograms was 63±13. The average Body Mass Index at third trimester was 30.5±5.6. Additional maternal profile is given in Table-I. Almost 81.3% (n= 366) of the women had a family income of less than 50,000 Pakistan Rupees (equal to US $ 387.5 per month) (Table-II). Another 63% (n= 282) had a distant or close family member suffering from Diabetes Mellitus. A worrying 17.1% (n=77) claimed of having a history of congenital abnormalities running in the family (Table-II). The prevalence of depression among women seeking antenatal care during their last trimester according to Goldberg Depression Scale was 40.89% (n=184) (Fig.1). On the other hand, 30.4% (n=137), 8.67% (n=39) and 1.78% (n=8) suffered from mild, moderate and severe depression respectively.

**Table-I:** Basic profile of study participants (n= 450).

Characteristics		N (%)
Gravidity	1	137(30.4%)
	2	121(26.9%)
	3	85(18.9%)
	4	61(13.6%)
	5 and more	46(10.2%)
Parity	0	169(37.6%)
	1	124(27.6%)
	2	87(19.3%)
	3	47(10.4%)
	4	23(5.1%)
Abortions	0	332(73.8%)
	1	86(19.1%)
	2	32(7.1%)
Number of Children	0	184(40.9%)
	1	123(27.3%)
	2	87(19.3%)
	3	36(8.0%)
	4	20(4.4%)
	5	13(2.9%)

**Table-II:** Associations of study participants with depression (n= 450).

Characteristics		Depression		P value

		Yes n(%)	No n(%)	
Family income	Less than 25000 PKR	70(15.6%)	107(23.8%)	0.359
	25000-50000 PKR	84(18.7%)	105(23.3%)	
	≥50000 PKR	30(6.6%)	54(12%)	
Passive Smoker	Yes	65(14.4%)	45(10%)	0.000^*^
	No	119(26.4%)	221(49.1%)	
Congenital Abnormalities in Family	Yes	31(6.9%)	46(10.2%)	0.902
	No	153(34.0%)	220(48.9%)	
Family History of Pre-eclampsia	Yes	65(14.4%)	55(12.2%)	0.001^*^
	No	119(26.4%)	211(47%)	
Family History of Depression	Yes	40(8.9%)	36(8.0%)	0.022^*^
	No	144(32%)	230(51.1%)	
Family History of Diabetes Mellitus	Yes	124(27.6%)	158(35.1%)	0.085
	No	60(13.3%)	108(24%)	

* . The Chi-square statistic is significant at the 0.05 level.

Almost 24.4% 9 (n=110) of the women during their last trimester of pregnancy for the antenatal checkup admitted to suffer from passive smoking, whereas the association between passive smoking and antenatal depression in last trimester was highly significant (p<0.01). About 26.6% (n=120) had a family history of pre eclampsia and the association between family history of preeclampsia and antenatal depression was highly significant (p<0.05). Almost 16.8% (n=76) of the women had a family history of depression and the association was significant (p<0.05) (Table-II).

## DISCUSSION

In a study conducted in Hyderabad, Sindh the frequency of depression during pregnancy (n=1.368) was found to be 18% using Aga Khan University Anxiety Depression Scale (AKUADS) at later half of second trimester.^18^ According to a study carried out in Punjab, prevalence of antenatal depression was 25%.^19^ In another descriptive cross sectional study conducted in District Chitral, the prevalence of depression was predicted to be 34% among 340 pregnant women.^20^ In 2007, a study from a tertiary care private hospital in Lahore assessed using Edinburgh Postnatal Depression Scale (EPDS) showed 42.7%(n=91) of women, scored above the cut-off for antenatal depression.^21^ The results correspond well with this study where the prevalence of depression among women seeking antenatal care during their last trimester according to Goldberg Depression Scale in Shalamar Hospital a private tertiary care institution of Lahore was 40.89% (n=184).

Passive smoking status was assessed by asking pregnant women if anyone in their house smokes around them during their current pregnancy if yes they were regarded as passive smoker? Passive smoking radically attributes to undesirable psychological wellbeing for women in antenatal phase, particularly due to their rigorous outcome of suicidal thoughts. Shu-Chuan Weng et al. in 2016 examined the association of exposure of passive smoking with suicidal thoughts, anxiety and depression in women from the first three months of pregnancy to the first month of puerperal phase in Taiwan and weighed against with women without antenatal passive smoking exposure. Exposure of passive smoking in pregnant women solitarily demonstrated elevated risks for suicidal thoughts in the second trimester (OR = 7.63; 95 % CI = 3.25–17.93) and third trimester (OR = 4.03; 95 % CI = 1.76–9.23).^22^ Women with exposure of passive smoking had a greater than before risk of depression (OR = 1.71; 95 % CI = 1.27–2.29). The results correspond well with this study where authors found out the association between passive smoking and antenatal depression in last trimester as highly significant (p < 0.01).

Al-Azri et al. (2016)carried out a cross-sectional study in Muscat, Oman on expectant Omani women greater and equal to 32 gestational weeks (n=959) attending primary care health center for scheduled regular antenatal care. It was established that antenatal depression was significantly associated with a positive history of depression among family members (*p* =0.019).^23^ The prevalence of family history of depression in our study was higher and almost 16.8% (n= 76) of the women had a family history of depression but the association was significant (p<0.05).

Rong Hu et al. in 2015 evaluated twelve studies with self-reported screening tools on antenatal depression and the risk of preeclampsia. Antenatal depression was significantly associated with both Caesarean delivery and preeclampsia (RR = 1.24; 95% CI, 1.14 - 1.35, and OR = 1.63, 95% CI, 1.32 - 2.02, respectively).^24^ Our study also evaluated that 26.6% (n= 120) had a family history of pre eclampsia and the association between family history of preeclampsia and antenatal depression was highly significant (p<0.05).

### Limitations of the study

It included convenience non probability sampling method. This study has been conducted in a tertiary hospital and the women outside of this tertiary hospital could not be screened for antenatal depression and relevant associations could not be estimated. Goldberg depression scale is a screening not a diagnostic tool. Consequently to confirm any case of depression, referral to a psychiatric panel should be done. It has also not been derived that antenatal depression might or might not proceed as postnatal depression after delivery. Another drawback of the present investigation is that the evaluation of depression was made only once during pregnancy, raising the issue about whether symptoms changed altogether after pregnancy for betterment or worsened.

## CONCLUSION

According to Goldberg Depression Scale, a significantly high percentage (41%) of women seeking antenatal care in their last trimester of pregnancy appearing in private tertiary care institution of Lahore suffered from Depression. The association between passive smoking and antenatal depression in last trimester was highly significant. Moreover, history of preeclampsia and family history of depression were also associated with antenatal depression.
